# HLA and Celiac Disease Susceptibility: New Genetic Factors Bring Open Questions about the HLA Influence and Gene-Dosage Effects

**DOI:** 10.1371/journal.pone.0048403

**Published:** 2012-10-31

**Authors:** Luz María Medrano, Bárbara Dema, Arturo López-Larios, Carlos Maluenda, Andrés Bodas, Natalia López-Palacios, M. Ángeles Figueredo, Miguel Fernández-Arquero, Concepción Núñez

**Affiliations:** 1 UGC de Inmunología, Hospital Clínico San Carlos, Instituto de Investigación Sanitaria del Hospital Clínico San Carlos (IdISSC), Madrid, Spain; 2 Servicio de Pediatría, Hospital Clínico San Carlos, Instituto de Investigación Sanitaria del Hospital Clínico San Carlos (IdISSC), Madrid, Spain; 3 Servicio de Aparato Digestivo, Hospital Clínico San Carlos, Instituto de Investigación Sanitaria del Hospital Clínico San Carlos (IdISSC), Madrid, Spain; University of Michigan Medical School, United States of America

## Abstract

Celiac disease (CD) is a chronic inflammatory disorder triggered after gluten ingestion in genetically susceptible individuals. The major genetic determinants are HLA-DQA1*05 and HLA-DQB1*02, which encode the DQ2 heterodimer. These alleles are commonly inherited in cis with DRB1*03∶01, which is associated with numerous immune-related disorders, in some cases contributing with a different amount of risk depending on the haplotype context. We aimed at investigating those possible differences involving DRB1*03∶01-carrying haplotypes in CD susceptibility. A family (274 trios) and a case-control sample (369 CD cases/461 controls) were analyzed. DRB1*03∶01-carrying individuals were classified according to the haplotype present (ancestral haplotype (AH) 8.1, AH 18.2 or non-conserved haplotype) after genotyping of *HLA-DRB1*, *-DQA1*, -*DQB1*, *-B8, TNF* -308, *TNF* -376 and the *TNFa* and *TNFb* microsatellites. We observe that the AH 8.1 confers higher risk than the remaining DRB1*03∶01-carrying haplotypes, and this effect only involves individuals possessing a single copy of DQB1*02. CD risk for these individuals is similar to the one conferred by inherit DQA1*05 and DQB1*02 in trans. It seems that an additional CD susceptibility factor is present in the AH 8.1 but not in other DRB1*03∶01-carrying haplotypes. This factor could be shared with individuals possessing DQ2.5 trans, according to the similar risk observed in those two groups of individuals.

## Introduction

Human leukocyte antigen (HLA) is a master piece in the pathogenesis of celiac disease (CD), as first evidenced by the strong genetic association existent between CD susceptibility and certain HLA alleles. This region, located on 6p21, contains hundreds of genes with immunological function and it is characterized by a high gene density and variability and an extensive linkage disequilibrium, which make difficult to pinpoint the causal variant/s. Despite this, CD can be considered a particular disease since the specific HLA alleles involved and their functional implication are well-established. The presence of the DQA1*05 and DQB1*02 susceptibility alleles implies the formation of the α and β chains of the HLA-DQ2 heterodimer, present in around 90–95% of CD individuals. This molecule shows high affinity for peptides resultant from incomplete gluten digestion, which bind and present to antigen specific T cells, triggering the intestinal inflammation prototypical of CD.

In most cases, DQA1*05 and DQB1*02 are encoded in the same chromosome (DQ2.5 cis) and appear in very strong linkage disequilibrium with DRB1*03∶01. In fact, this allele was first associated with CD risk [1]. The DQA1*05, DQB1*02 and DRB1*03∶01 alleles can be present in two different haplo-specific contexts and constitute the so-called ancestral haplotypes (AH) 8.1 and AH 18.2; they can also be found within non-specific allelic combinations and constitute other less frequent haplotypes (hereafter called non-conserved haplotypes). The DRB1*03∶01 allele, and consequently, DRB1*03∶01 haplotypes, have been associated to numerous immune-mediated disorders, as type 1 diabetes, multiple sclerosis or selective IgA deficiency, among many others; in some cases with the different DRB1*03∶01 haplotypes showing a differential contribution to disease risk [2], [3]. A differential behaviour between AH 18.2 and AH 8.1 has also been described in CD [4], [5], although no relevance has been given to this observation. The DQA1*05 and DQB1*02 alleles can also be inherited in trans, each encoded in one chromosome from each parent (DQ2.5 trans).

HLA influence on CD susceptibility shows a dose effect. Individuals can be classified in high or intermediate CD risk according to the number of DQA1*05- and DQB1*02-carrying alleles. Homozigosity for DQ2.5 cis and heterozigosity for DQ2.5 cis with a chromosome possessing a second DQB1*02 allele (DQ2.2) confer the highest risk to develop CD. Heterozigosity for DQ2.5 cis in individuals with a single copy of DQB1*02 (non-DQ2.2) or presence of DQ2.5 trans confer intermediate risk.

Additionally to the molecule DQ2.5, the influence of HLA-DQ8 (genetically DQA1*03, DQB1*03∶02) on the disease is already known. This molecule is present in almost all the CD patients without DQ2.5. However, the genetic influence of the HLA region in CD is not limited to the factors coding DQ2 or DQ8, and several works have attempted to discover new susceptibility factors without much success (see [6] for review). Some variants in the *TNF* gene have been suggested as DQ2 independent factors for CD susceptibility, even as the responsible factors for the additional risk present on the AH 8.1. [7]. Last years have witnessed a spectacular increase in the knowledge of the genetic basis of CD, favoured by development of genome wide association studies (GWAS), but these works have not added new information about the HLA contribution because they have been mainly focused on the influence of genes outside this region.

We aimed at investigating the additional genetic contribution to CD susceptibility lying on the HLA region, by focusing in the possible differential contribution of the different DRB1*03∶01-carrying haplotypes.

## Materials and Methods

### Ethics Statement

This study was approved by the ethical committee (CEIC) of Hospital Clínico San Carlos. Samples were obtained after obtaining written informed consent. For children, the informed consent was signed by their parents or legal guardian.

### Subjects

A total of 274 trios composed for both parents and the affected child and a case-control series consisting of 369 independent CD patients and 461 ethnically matched healthy controls were studied. CD patients were diagnosed following the European Society for Pediatric Gastroenterology, Hepatology, and Nutrition (ESPGHAN) [8], 97% are positive for HLA-DQ2 and/or HLA-DQ8. Controls correspond mainly to blood donors and laboratory staff. CD samples were consecutively collected in two centres of the same region (Hospital La Paz and Hospital Clínico San Carlos, Madrid, Spain) and controls were collected at the Hospital Clínico San Carlos. All samples correspond to unrelated Spanish white individuals.

### Genotyping

DNA was extracted from fresh peripheral blood leukocytes by a “salting out” procedure. All samples were genotyped for *HLA-DRB1*, *-DQA1* and -*DQB1* by PCR-SSOP (Polymerase Chain Reaction-Sequence Specific Oligonucleotide Probe). The different DRB1*03∶01 haplotypes were assessed by additional genotyping of the *TNF* single nucleotide polymorphisms (SNPs) -308 (rs1800629) and -376 (rs1800750) and the microsatellites *TNFa* and *TNFb*; those polymorphisms were typed as previously described [9], [10]. The presence of the *HLA-B8* allele was tested by TaqMan technology using the tag SNPs rs6457374 and rs2844535 (Applied Biosystems Inc., Foster City, CA, USA).

We defined the AH 8.1 according to the simultaneously presence of DRB1*03∶01, DQA1*05∶01, DQB1*02∶01, TNF -308A, TNFa2b3 and B*8. AH 18.2 was defined according to carriage of DRB1*03∶01, DQA1*05∶01, DQB1*02∶01, TNF -376A and *TNFa1b5*. Haplotypes with all the remaining allelic combinations in those loci or markers were designed as non-conserved haplotypes. The *TNF* markers studied were selected because, in DRB1*03∶01 subjects, they are haplo-specific for AH 18.2 or AH 8.1.

### Statistical Analysis

HLA haplotypes were deduced directly from the pedigree for patients used in the family study. In cases and controls, the EM (Expectation-Maximization) algorithm implemented in the Arlequin software was used to estimate haplotype frequencies.

The transmission disequilibrium test (TDT) was used to analyse the preferential transmission of one haplotype over the others when analysing family data. This test uses only information provided by heterozygous parents.

Comparisons between groups were performed with the chi-square test using the statistical package EpiInfo v5.00 (CDC, Atlanta, USA). Heterogeneity between haplotype groups was evaluated with Review Manager (RevMan) 5.0 software (Copenhagen: The Nordic Cochrane Centre, The Cochrane Collaboration, 2008).

## Results

We studied 274 trios to investigate the possibility of a differential transmission of the different DRB1*03∶01 haplotypes to the affected child (Table 1). DRB1*03∶01 is always preferentially transmitted, independently of its haplotype context (see TDT results, in Table 1). However, the distortion in the transmission of this allele is significantly higher when it is present in the AH 8.1, compared with its presence in the remaining DRB1*03∶01-containing haplotypes (p = 8.7*10^−4^ vs. AH 18.2; p = 2.4*10^−4^ vs. non-conserved haplotypes). AH 18.2 and non-conserved haplotypes show a similar preferential transmission to offspring (p = 0.99). These differences are also observed when considering the haplotype transmission from the DRB1*03∶01 homozygous parents (composed by different DRB1*03∶01 haplotypes) included in the 274 families (Table 2).

**Table 1:** Transmission data of the diverse DRB1*03∶01-containing haplotypes in the 274 families studied and TDT results.

DRB1*03∶01 HAPLOTYPES	T	U	TDT
1. All	268	88	p<10^−10^
2. AH 8.1	118	18	p<10^−10^
3. AH 18.2	58	27	p = 2.2*10^−4^
4. Non-conserved	92	41	p = 4.9*10^−6^
5. Non-AH 8.1	150	68	p = 5.3*10^−9^

T = transmitted; U = untransmitted; TDT = transmission disequilibrium test.

Non-AH 8.1 includes AH18.2 and non-conserved haplotypes.

TDT results are calculated after excluding homozygous parents (5 for AH 8.1, 5 for AH 18.2 and 7 for DRB1*03∶01 non-conserved haplotypes).

Haplotype comparisons: 2 vs. 3: p = 0.00087 OR = 3.05 95% CI 1.48–6.33; 2 vs. 4: p = 0.00024 OR = 3.06 95% CI 1.59–5.94; 3 vs. 4: p = 0.99 OR = 1.00 95% CI 0.54–1.88; 2 vs. 5: p = 7.8*10^−5^ OR = 3.06 95% CI 1.67–5.65.

**Table 2:** Transmission data of the diverse DRB1*03∶01-containing haplotypes from DRB1*03∶01 homozygous parents (N = 27).

HAPLOTYPE COMPOSITION	N	T	TDT
AH 8.1 - AH 18.2	6	6 AH 8.1, 0 AH 18.2	p = 0.016
AH 8.1 - Non-conserved	10	7 AH 8.1, 3 Non-conserved	p = 0.17
AH 18.2 - Non-conserved	11	4 AH 18.2, 7 Non-conserved	p = 0.27
AH 8.1– Non-AH 8.1	16	13 AH 8.1, 3 Non- AH 8.1	p = 0.011

T = transmitted; TDT = transmission disequilibrium test.

We wanted to validate this observation in a case-control sample (Table 3). Since no differences were observed between AH 18.2 and non-conserved haplotypes in the family data, they were combined in subsequent analysis (and called non-AH 8.1). As already known, DRB1*03∶01 overall appears at significantly higher frequency in CD patients than in controls: 45% vs. 14%, respectively (OR = 4.97 95% CI 3.90–6.34, p<10^−7^); but we additionally show that this case-control difference is higher when considering only the AH 8.1 (OR = 6.53 95% CI 4.47–9.56, p<10^−7^).

**Table 3:** Frequency and comparison of the diverse DRB1*03∶01 haplotypes in celiac disease (CD) patients (2N = 738) and controls (2N = 922), overall and classified according to the different HLA CD risk categories.

DRB1*03∶01 HAPLOTYPES	CD		CONTROLS		CD vs. CONTROLS			HETEROGENEITYAH 8.1 VS. NON-AH 8.1		
	2N	%	2N	%	p		OR (95% CI)	p		I^2^
ALL DRB1*03∶01										
All	330	44.7	129	14.0	<10^−7^	4.97 (3.90–6.34)				
AH 8.1	141	19.1	42	4.6	<10^−7^	6.53 (4.47–9.56)			0.06	71%
Non-AH 8.1	189	25.6	87	9.4	<10^−7^	4.22 (3.16–5.65)				
**DQ2.5 cis+DQ2.5 cis**										
All	74	10.0	16	1.7	<10^−7^	8.99 (5.03–16.28)				
AH 8.1	30	4.1	6	0.65	<10^−7^	9.72 (3.93–28.74)			0.82	0%
Non- AH 8.1	44	6.0	10	1.1	<10^−7^	8.55 (4.10–18.31)				
**DQ2.5 cis+DQ2.2**										
All	104	14.1	13	1.4	<10^−7^	15.55 (8.39–29.37)				
AH 8.1	40	5.4	6	0.7	<10^−7^	12.96 (5.39–37.64)			0.60	0%
Non- AH 8.1	64	8.7	7	0.8	<10^−7^	17.77 (8.03–46.30)				
**DQ2.5 cis+non-DQ2.2**										
All	152	20.6	100	10.8	<10^−7^	2.95 (2.21–3.94)				
AH 8.1	72	9.8	30	3.3	<10^−7^	4.66 (2.94–7.44)			0.009	85%
Non-AH 8.1	80	10.8	70	7.6	3.2*10^−6^	2.22 (1.56–3.17)				

% are referred to the total number of haplotypes.

Non-AH 8.1 includes AH 18.2 and non-conserved haplotypes.

DQ2.5 cis = DQB1*02∶01-DQA1*05∶01; DQ2.2 = DQB1*02∶02-DQA1*02∶01.

In CD, it is well established the existence of a dose effect, what implies the existence of different CD risk categories attending to their HLA constitution. We investigated this differential risk contribution of DRB1*03∶01-containing haplotypes in those categories and found that only in those individuals carrying a single copy of the DQB1*02 allele (individuals DQ2.5 cis + non-DQ2.2 in Table 3), the presence of AH 8.1 confers additional risk.

When considering the HLA risk categories according to gene dosage effects, carriage of the DQ2 molecule in individuals with a single copy of the DQB1*02 allele is considered as conferring intermediate CD risk. A similar risk is conferred by the presence of DQ2.5 trans, although some groups reported this to be an intermediate higher risk group [11]. We compared CD risk in carriers of DQ2.5 trans (48 individuals out of 369 patients and 21 out of 461 controls) to CD risk in carriers of DQ2.5 cis with and without the AH 8.1 and we observed that DQ2.5 trans confers similar risk than DQ2.5 cis with AH 8.1 (heterogeneity: p = 0.91, I^2^ = 0%) and significantly higher risk than DQ2.5 cis with non-AH 8.1 haplotypes (heterogeneity: p = 0.09, I^2^ = 64%).

Finally, we investigated the possibility that the similar risk conferred by DQ2.5 trans and DQ2.5 cis with AH 8.1 was due to the presence of a common susceptibility factor. In most cases, carriers of the molecule DQ2.5 trans are genetically characterized by being heterozygous DQB1*03∶01-DQA1*05∶05/DQB1*02∶02-DQA1*02∶01 (serologically DR5/DR7, terms also used hereafter for simplification purposes). We used genotype data corresponding to 6,769 SNPs located in the HLA (29.96–33.19 Mb interval), which were previously obtained in a subset of our Spanish samples (more than 500 CD patients and 300 controls) in the context of the Immunochip Project (http://www.immunobase.org). We selected all the homozygous individuals for AH 8.1, AH 18.2, DR5 (DQB1*03∶01-DQA1*05∶05) and DR7 (DQB1*02∶02-DQA1*02∶01) and looked for a chromosomal region shared between AH 8.1 and DR5 (DQB1*03∶01-DQA1*05∶05) or AH 8.1 and DR7 (DQB1*02∶02-DQA1*02∶01) but not common to AH 18.2. However, no regions emerged after that search. Note that this result could be affected by the low number of available homozygous individuals, mainly DR5 (DQB1*03∶01-DQA1*05∶05) and DR7 (DQB1*02∶02-DQA1*02∶01) (3 and 4, respectively).

## Discussion

Our results evidence the presence of an additional susceptibility factor to CD in the HLA region, which is linked to AH 8.1. This factor only increases susceptibility when appearing in individuals carrying a single copy of the DQB1*02 allele (DQ2.5 cis non-DQ2.2) and split up the HLA intermediate risk group into two groups: one with higher intermediate risk, which is composed of carriers of the molecule DQ2.5 cis encoded by AH 8.1 and carriers of the molecule DQ2.5 trans; and one with lower intermediate risk, which is composed of carriers of the molecule DQ2.5 cis encoded by either AH 18.2 or non-conserved DRB1*03∶01 haplotypes.

The well-known contribution of the DQ2 molecule to CD pathogenesis is genetically based on carriage of DQA1*05 and DQB1*02. These two alleles are commonly present in the small segment identical by descent among DRB1*03∶01-containing haplotypes. However, outside that shared segment, the divergence between DRB1*03∶01 haplotypes do not differ from that found between disparate haplotypes [12]; therefore, a susceptibility factor located there is not expected to be present in all DRB1*03∶01 haplotypes. On the other hand, previous studies suggested a close evolutionary relationship among DRB1*03∶01-containing haplotypes, DR5 and DR7 (DQB1*03∶01-DQA1*05∶05 and DQB1*02∶02-DQA1*02∶01) [13], which could explain the existence of a common susceptibility factor between AH 8.1 and one of those haplotypes. No definitive conclusion can de drawn from our data due to the low number of chromosomes compared. Further analysis including higher sample size is mandatory.

The analysis of the HLA region in GWAS showed, besides the expected peak corresponding to DQ2.5 cis, two SNPs associated to CD risk, both located within or adjacent to *HLA-DQA1* and *HLA-DQB1* [14]. This is in accordance with our results, which suggest a risk factor common to DRB1*03∶01-containing haplotypes and DR5 or DR7 (DQB1*03∶01-DQA1*05∶05 or DQB1*02∶02-DQA1*02∶01), because those are the regions that they shared.

One intriguing issue emerging from our study is why the additional risk factor present in the AH 8.1 does not seem to influence on CD susceptibility when it appears in individuals carrying a second copy of the DQB1*02 allele. In CD, T cell stimulation due to gluten-derived peptides depends on the number and type of HLA-DQ2 molecules expressed. DQ2.5 molecules can bind a high repertoire of gluten peptides, but only a restricted subset is bound to DQ2.2 molecules, which reduce the immunogenicity of DQ2.2. Additionally, the number of these DQ molecules is also a relevant factor in T cell stimulation and this depends on the number of specific alleles in *DQA1* and *DQB1* loci, which determines the possible αβ-chain combinations constituting the DQ heterodimers [15]. As a matter of fact, all HLA-DQ molecules are identical in HLA-DQ2.5 homozygous individuals, which can bind a very high repertoire of gluten peptides and confer the highest CD risk. It could be speculated that in such scenario an additional susceptibility factor has null or limited possibility to increase risk. By the contrary, in HLA-DQ2.5 cis individuals (without a second copy of DQB1*02, i.e., non-DQ2.2), only one of the four possible αβ combinations constitutes an HLA-DQ2.5 molecule and therefore the presence of a genetic factor which increases immunogenicity against gluten derived peptides could have a relevant impact in increasing CD risk.

The HLA dose effect is also influenced by differences in the kinetic stability of the interaction between HLA molecules and gluten derived peptides, key factor for development of T cell responses against gluten [16], [17]. For most peptide ligands, DQ2.5 shows higher binding stability than DQ2.2. The risk variant present in the AH 8.1, and presumably in individuals possessing DQ2.5 trans molecules, could affect the kinetic stability of the interaction HLA-gluten either increasing the number of gluten derived peptides which can bind with high affinity or increasing the binding stability of peptides previously recognised. Bodd *et al* [17] claimed that T-cell epitopes must be assessed and characterized in the context of the HLA molecules expressed by the T-cell donor and underlined the relevance that this could have for future peptide-based vaccines. According to that, it would be interesting to establish a comparison of the HLA-DQ molecules present in individuals DQ2.5 cis with AH 8.1, DQ2.5 cis with non-AH 8.1 haplotypes and DQ2.5 trans. Differences in their binding gluten peptides would imply that peptide-based vaccines should look at those individuals differentially.

Much more work deserves this field, with several open questions as which is the causal variant lying on the AH 8.1 and which are their specific functional implications.
